# Epidemiological and Genomic Characteristics of *bla*_CTX-M_ and *bla*_NDM_‐Producing *K. pneumoniae* From Dairy Cows in Henan Province, China

**DOI:** 10.1155/tbed/5331014

**Published:** 2026-05-30

**Authors:** Yiming Feng, Wenfeng Li, Mengjuan Ma, Yajun Zhai, Hua Wu, Jianhua Liu, Gongzheng Hu, Dandan He, Yushan Pan

**Affiliations:** ^1^ College of Veterinary Medicine, Henan Agricultural University, Zhengzhou, Henan, China, henau.edu.cn

**Keywords:** antimicrobial resistance dissemination, bovine-origin *Klebsiella pneumoniae*, carbapenem resistance, extended-spectrum β-lactamase

## Abstract

Extended‐spectrum β‐lactamase (ESBL) and carbapenemase‐producing *Klebsiella* spp., particularly *K. pneumoniae*, are emerging threats in both clinical and community settings. To investigate the epidemiology, phylogenetics, and antimicrobial resistance of bovine‐origin strains, 174 bacterial isolates were obtained from 180 mastitic milk samples across eight dairy farms in Henan Province. Sixty‐five isolates were identified as *Klebsiella* spp., including 61 *K. pneumoniae*, 2 *K. quasipneumoniae*, 1 *K. oxytoca*, and 1 *K. michiganensis*. Phylogenetic analysis assigned most isolates to the KpI phylogroup, and multilocus sequence typing revealed 24 sequence types, with ST107 and ST1083 predominating. SNP‐based phylogeny indicated two major clades with evidence of clonal spread. Compared with human hypervirulent reference strains, bovine isolates were genetically distinct, lacking key virulence genes *rmpA*, *iuc*, and *iro*, while only a minority carried *ybt*. Among the 65 *Klebsiella* isolates, 31 (47.7%) were ESBL producers, mainly carrying *bla*_CTX-M−3_ or *bla*_CTX-M−15_, one *K. pneumoniae* isolate harbored the carbapenemase‐encoding gene *bla*_NDM−1_. ESBL producers showed >90% resistance to β‐lactams but remained largely susceptible to ciprofloxacin and gentamicin, with 87.1% displaying multidrug resistance. Co‐occurrence of multiple resistance and virulence determinants was observed, and the dissemination of *bla*_CTX-M_ genes was strongly associated with the mobile element IS*Ecp1*. Plasmid analysis revealed that *bla*_CTX-M_ genes were primarily located on IncFIB(K) plasmids, while the *bla*_NDM−1_‐positive isolate carried a transferable IncC2 plasmid. SNP analysis further supported clonal dissemination among isolates with identical STs. These findings demonstrate that dairy cattle serve as important reservoirs of multidrug‐resistant *Klebsiella*, where resistance is driven by both clonal transmission and plasmid‐mediated horizontal transfer. This highlights potential risks for animal health, antimicrobial stewardship, and zoonotic transmission to humans.

## 1. Introduction

The rapid emergence and spread of antibiotic resistance in pathogens are a growing global concern, particularly in agricultural settings. This poses significant risks not only to animal health but also to public health as resistant bacteria can transfer between animals and humans. Mastitis is a common disease in dairy cows that results in substantial economic losses to dairy farming and constitutes a potential threat to public health. Bacterial infections are a major cause of mastitis in cows [1], with major pathogens including *S. agalactiae*, *S. dysgalactiae*, *S. uberis*, *K. pneumoniae*, *S. aureus*, *E. coli*, and others [2]. Several studies indicated that *E. coli* is more prevalent among ESBL‐producing strains from milk [3, 4]. In contrast, reports concerning ESBL‐producing *K. pneumoniae* from milk samples remain relatively scarce. Notably, the World Health Organization (WHO) ranks *K. pneumoniae* as the third most critical priority pathogen after *E. coli* and *S. aureus* [5], highlighting the importance of further research on its resistance characteristics.

In recent years, the overuse and misuse of antimicrobial agents in human healthcare, animal husbandry, and agriculture have accelerated the emergence of antimicrobial resistance [6]. The WHO has identified several high‐risk antimicrobial resistance genes (ARGs), particularly those encoding extended‐spectrum β‐lactamases (ESBLs) and carbapenemases, as critical public health concerns due to their ability to inactivate clinically essential antibiotics [7]. Among these ARGs, *bla*_CTX-M_ and *bla*_NDM_ are particularly concerning. The *bla*_CTX-M_ gene encodes ESBLs that confer resistance to cephalosporins, while *bla*_NDM_ mediates resistance to carbapenems—often regarded as last‐resort antibiotics. The presence of these genes in *Enterobacteriaceae*, especially in *E. coli* and *K. pneumoniae*, represents a serious threat to both human and animal health due to their strong association with multidrug resistance (MDR) [8].

This global threat is mirrored in the dairy sector. Although good husbandry practices and milking hygiene are the primary strategies to prevent bovine mastitis, antibiotic treatment—especially with third‐generation cephalosporins such as ceftiofur—is still common in clinical settings [9]. Alarmingly, many of these antibiotics, including cephalosporins and carbapenems, are classified as “Reserve” drugs in the WHO’s “Access, Watch, Reserve” (AWaRe) list due to their importance in treating severe human infections [10]. Their extensive use in livestock has contributed to the rising prevalence of ESBL‐ and carbapenemase‐producing bacteria. For instance, Gelalcha et al. [11] reported that 9.9% (57/572) of environmental, fecal, and feed samples from 14 dairy farms in Tennessee tested positive for ESBL‐producing *Klebsiella* spp., while in northern Xinjiang, China, Huang et al. [12] found a 19.5% prevalence (149/766) of ESBL‐producing *E. coli*. These findings highlight that dairy farms can serve as significant reservoirs for resistant pathogens, further emphasizing the need for targeted surveillance and control measures.

In this context, the present study aims to investigate the prevalence, resistance patterns, and molecular epidemiology of *bla*_CTX-M-_ and *bla*_NDM_‐producing *Klebsiella* strains isolated from mastitic milk samples in dairy farms in Henan Province, China. The study focuses on elucidating the epidemiological characteristics and resistance patterns of *Klebsiella* in dairy cow mastitis. By characterizing the genetic determinants and transmission pathways of these resistance genes, this research seeks to provide valuable insights into the dissemination of multidrug‐resistant *Klebsiella* and ultimately support the development of effective strategies to control mastitis and mitigate the broader spread of antimicrobial resistance in the environment.

## 2. Materials and Methods

### 2.1. Bacterial Isolates and Identification

A total of 180 milk samples were collected from cows with mastitis across 8 dairy farms in Henan Province. The samples were incubated in LB broth at 37°C for 16–18 h and then plated onto MacConkey agar. The isolated strains were purified and identified through 16S rRNA gene sequencing. For the mastitis cases at these farms, the commonly used antibiotics were, in descending order of usage, ceftiofur, cefquinome, oxytetracycline, tetracycline, and amoxicillin. This usage pattern may contribute to the selection pressure driving antimicrobial resistance among the isolated strains.

### 2.2. PCR Amplification

PCR was carried out to amplify the resistance genes *tet*(A), *floR*, *bla*_NDM_, *bla*_OXA_, and *bla*_CTX-M_ using the specific primers listed in Table S1. The reaction conditions, including annealing temperatures and cycling parameters, were optimized for each target gene. PCR products were analyzed by agarose gel electrophoresis and sequenced for confirmation.

### 2.3. Antimicrobial Susceptibility Testing

The minimum inhibitory concentrations (MICs) of 14 antimicrobial agents against 31 ESBL‐producing *Klebsiella* strains were determined using the microdilution broth method. The tested antimicrobial agents included ciprofloxacin, florfenicol, amikacin, enrofloxacin, colistin, cefuroxime, cefotaxime, ampicillin, doxycycline, gentamicin, cefoxitin, ceftazidime, tigecycline, and meropenem. *E. coli* ATCC 25922 was used as a quality control strain. The results were interpreted according to the Clinical and Laboratory Standards Institute (CLSI) guidelines [13]. For tigecycline and florfenicol, breakpoints were interpreted according to the FDA and EUCAST criteria, respectively (https://mic.eucast.org/).

### 2.4. Conjugation Assays

The *bla*_NDM_‐positive *K. pneumoniae* HN148 strain was used as the donor, while rifampin‐resistant *E. coli* C600 served as the recipient to assess the transferability of the *bla*_NDM−1_ gene through conjugation. Transconjugants were selected on MacConkey agar supplemented with meropenem (1 mg/L) and rifampin (450 mg/L). The conjugation frequency was calculated based on the number of transconjugants per recipient.

### 2.5. Whole Genome Sequencing (WGS) and Data Analysis

The genomic DNA of all *Klebsiella* isolates was extracted using the QIAamp DNA Mini Kit (QIAGEN, Hilden, Germany). WGS was performed using the Illumina HiSeq 2000 platform. For strain HN148, additional WGS was performed using the Oxford Nanopore Technologies MinION platform. Sequencing reads, including short‐read and long‐read data, were assembled with Unicycler 0.4.4 with the hybrid assembly strategy.

The sequence was initially annotated using the RAST server (http://rast.nmpdr.org) and corrected manually based on NCBI (https://www.ncbi.nlm.nih.gov/). ARGs, plasmid replicant types, and ST types were identified using ResFinder and PlasmidFinder software on the CGE server (https://cge.cbs.dtu.dk/services/). Insertion sequences (ISs) were determined using the ISfinder (https://isfinder.biotoul.fr/). MOB‐suite (mob_recon) was used to predict the plasmid backbones carrying *bla*_CTX-M_ [14]. The putative coding sequences (CDSs) were identified using the ORF Finder program (http://www.ncbi.nlm.nih.gov/projects/gorf/). Kleborate and Kaptive were used to identify the virulence factors, capsule synthesis locus (K‐locus), and lipopolysaccharide (O) types in *Klebsiella* species [15, 16]. Clonal groups (CGs) of *K. pneumoniae* were assigned based on the sequences of seven highly conserved housekeeping genes (*gapA*, *infB*, *mdh*, *pgi*, *phoE*, *rpoB*, and *tonB*), following the scheme of the Institut Pasteur MLST database (https://bigsdb.pasteur.fr/). Kleborate was used in conjunction to facilitate sequence analysis and CG designation. In addition, *gyrA* and *parC* gene sequences were analyzed to determine phylogroups (KpI, KpII, and KpIII) [17]. Core genes of the bacterial genome were extracted and aligned using Roary [18]. Then, FastTree was used to construct a phylogenetic tree based on single nucleotide polymorphisms (SNPs) of the core genome [19]. Illumina reads from each isolate were processed using the Snippy pipeline (v4.6.0). Reads were mapped to the selected reference genome (HN82) with BWA‐MEM (as implemented in Snippy), and variants were called with FreeBayes using default parameters. Core‐genome SNPs were extracted with snippy‐core to generate a core SNP alignment, and pairwise core‐genome SNP distances were calculated from this alignment using snp‐dists [20]. Graph networks describing the co‐occurrence of ARGs and ISs were generated using Gephi [21]. We determined a correlation coefficient of “1” when specific ARGs and IS elements were present in a contig. The correlation coefficient reflects the number of contig groups co‐carrying a particular ARG and an IS. Phylogenetic trees were visualized and modified using iTOL v6. A circular plasmid map was created using the Easyfig tool [22].

### 2.6. Accession Number

The WGS data of strains HN148 can be accessed through the BioProject ID PRJNA1221095. Short‐read sequencing data have been deposited into GenBank under the BioProject IDs PRJNA1224793 and PRJNA1415966.

## 3. Results

### 3.1. Genotypic Characterization of 65 *Klebsiella* Strains

A total of 65 *Klebsiella* strains were isolated from 180 clinical mastitis milk samples collected from 8 dairy farms in Henan Province; in total, 192 bacterial isolates were recovered from these 180 samples. Among these, 61 were identified as *K. pneumoniae*, 1 as *K. oxytoca*, 2 as *K. quasipneumoniae*, and 1 as *K. michiganensis*. PCR analysis revealed that the detection rates of the *bla*_CTX-M_, *tet*(A), and *floR* genes were 47.7% (31/65), 46.2% (30/65), and 35.4% (23/65), respectively, with *bla*_NDM−1_ being detected in only 1.5% (1/65) of the isolates. Among these, 17 isolates carried *tet*(A), *floR*, and *bla*_CTX-M_ simultaneously. Of the 31 ESBL‐producing isolates, 25 (80.6%) harbored two or more β‐lactamase genes, primarily *bla*_CTX-M_ (*n* = 31) and *bla*_TEM_ (*n* = 25) (Figure S1). Additionally, *bla*_SHV−27_ was identified in eight isolates, whereas *bla*_OXA_ was not detected. Within the *bla*_CTX-M_ family, the most prevalent group was *bla*_CTX-M−1_, followed by *bla*_CTX-M−9_. No *bla*_CTX-M−2_, *bla*_CTX-M−8_, or *bla*_CTX-M−25_ groups were detected. Further subtyping of the *bla*_CTX-M−1_ group revealed that *bla*_CTX-M−3_ and *bla*_CTX-M−15_ were the dominant variants, while the *bla*_CTX-M−9_ group primarily consisted of *bla*_CTX-M−14_ and *bla*_CTX-M−27_. No rare ESBL types were identified among the ESBL‐positive *K. pneumoniae* isolates.

Based on *gyrA* gene sequence analysis, 61 strains of *K. pneumoniae* and 2 strains of *K. quasipneumoniae* were assigned to phylogroups KpI and KpII, with the majority (93.8%, 61/65) belonging to KpI. MLST further divided the 61 KpI *K. pneumoniae* strains into 24 sequence types (STs), including predominant types such as ST107 and ST1083 (each *n* = 6), ST37 and ST716 (each *n* = 5), and ST116, ST219, ST229, and ST6 (each *n* = 4). The remaining strains were assigned to 10 rare STs, each represented by a single isolate—ST110, ST225, ST236, ST29, ST337, ST35, ST3687, ST4459, ST4467, and ST784—as well as 12 variant STs. Kleborate analysis revealed 23 capsule locus (KL) types among these strains, with KL132 (11.5%), KL114, KL110, and KL101 (each 9.8%) being the most common. The O‐antigen types were classified into six groups, with O1 being the most frequent (47.5%), followed by O3/O3a (19.7%), O2a (8.2%), O2afg (8.2%), O3b (4.9%), and O4 (3.3%), while 8.2% of strains had untypeable O‐loci (Figure S2). CG typing grouped the 61 KpI strains into 17 defined CGs—including CG10348, CG107, CG116, CG219, CG716, and CG6, among others—and 16 strains belonged to undefined CGs (Figure S3).

### 3.2. Phylogenetic Analysis of 61 Bovine‐Origin *K. pneumoniae* Strains

Based on SNP analysis of 61 bovine‐origin *K. pneumoniae* strains, phylogenetic analysis revealed two major clades (Clade I and Clade II) (Figure S4). Clade I comprised strains associated with ST784, ST29, ST5477, ST116, ST35, ST4467, ST6, ST110, ST229, ST219, and ST107. Closely related strains in this clade typically differed by 0–7 SNPs; for example, HN101 and HN113 showed 0 SNP differences, while HN107 and HN83 differed by 1 SNP. Within Clade I, the predominant ST107 and ST219 subclusters exhibited very low SNP distances of 0–3, suggesting clonal expansion within these lineages. Clade II included strains associated with ST236, ST3687, ST891‐2LV, ST6093, 190‐2LV, ST716, ST225, ST4680‐2LV, ST4459, ST37, and ST1083. This clade showed greater internal diversity, with longer average branch lengths and larger SNP differences within these groups. Notably, strains related to ST37 were distributed across multiple subclusters; for instance, HN145 and HN186 differed by 4 SNPs, while HN197 and HN200 had no SNP differences.

**Figure 1:**
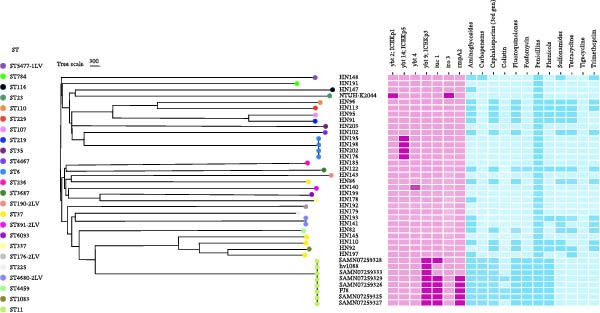
Comparison of different sequence types in bovine‐origin *K. pneumoniae* with those in hypervirulent ST23 and hypervirulent carbapenem‐resistant ST11 strains. The WGS data of the NTUH‐K2044 strain and eight ST11 CR‐HvKP isolates are available under BioProject IDs PRJDA21069 and PRJNA391211.

Phylogenetic analysis revealed that bovine‐origin *K. pneumoniae* strains and human‐origin hypervirulent reference strains (including one ST23‐type NTUH‐K2044 strain and eight ST11‐type carbapenem‐resistant hypervirulent *K. pneumoniae* (CR‐HvKP) strains) belonged to distinct branches on the phylogenetic tree, with substantial SNP differences of ~25,000 (Figure 1). Regarding virulence gene distribution, the hypervirulent ST23‐type NTUH‐K2044 carried the *ybt2*, *iro3*, and *rmpA* genes, while the hypervirulent ST11‐type CR‐HvKP carried the *rmpA*, *iuc1*, and *ybt9* genes. Among the bovine‐origin strains, only a subset carried the *ybt* gene; the majority lacked the *ybt*, *iuc*, and *iro* genes, and none carried the *rmpA* gene. The antimicrobial resistance profiles showed that the hypervirulent ST23‐type NTUH‐K2044 was susceptible to all drugs except penicillins. In bovine‐origin *K. pneumoniae*, strains carrying the *ybt* gene were susceptible to most antibiotics. However, strains lacking the *ybt* and *iuc* genes (such as ST229, ST107, and ST219) exhibited resistance to multiple drug classes, including aminoglycosides, third‐generation cephalosporins, fluoroquinolones, tetracyclines, chloramphenicol, and sulfonamides. Additionally, the hypervirulent ST11‐type CR‐HvKP demonstrated resistance to multiple agents, such as aminoglycosides, carbapenems, cephalosporins, colistin, fluoroquinolones, and fosfomycin. To assess the relatedness of bovine ST37 isolates to human‐derived ST37 lineages, we compared our bovine ST37 genomes with 490 publicly available human ST37 *K. pneumoniae* genomes. Pairwise core‐genome SNP analysis showed that bovine‐human ST37 comparisons differed by 955‐14,313 SNPs (minimum, 955; Figure S5).

### 3.3. Antimicrobial Susceptibility and Resistance Profiles of 65 *Klebsiella* Isolates

MICs for 14 antibiotics across seven classes were determined by the microdilution method for 65 *Klebsiella* isolates. Resistance was highest to ampicillin (87.69%), followed by cefuroxime, cefotaxime, and doxycycline (each 47.69%). By contrast, resistance to ciprofloxacin, colistin, gentamicin, cefoxitin, and tigecycline remained low (4.62%–9.23%). All isolates were susceptible to amikacin, and only a single isolate (HN148) showed resistance to meropenem (Figure S6). The resistance spectrum across the 65 isolates ranged from complete susceptibility to all tested agents to resistance to 10 antibiotics (Table S2). Single‐drug resistance was observed in 40.0% (26/65) of isolates, and 47.69% were resistant to three or more antibiotics. The dominant MDR profiles (4 isolates each) were enrofloxacin–cefuroxime–cefotaxime–ampicillin–doxycycline–ceftazidime and florfenicol–enrofloxacin–cefuroxime–cefotaxime–ampicillin–doxycycline. Resistance to six and seven antibiotics accounted for the largest proportions (16.92% and 10.77%, respectively; Figure S7), whereas resistance to 10 antibiotics was rare (1.54%).

### 3.4. Molecular Characteristics of ESBL‐Producing *Klebsiella* Isolates

**Figure 2:**
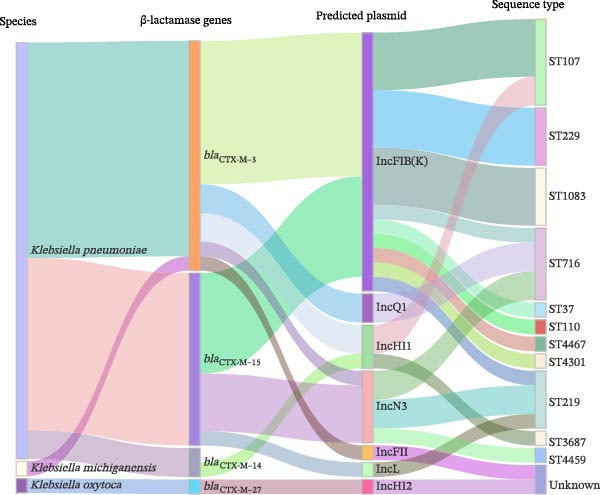
Gene mapping and correlation analysis of key traits of 31 ESBL‐producing *Klebsiella* strains. Sankey diagram illustrating the relationships between species, β‐lactamase gene subtypes, gene localization, and ST types of ESBL‐producing *Klebsiella*.

WGS results showed that ESBL‐producing *K. pneumoniae* strains belonged to 11 types, including ST4459, ST716, ST1083, ST107, ST219, ST110, ST229, ST4467, ST37, ST4301, and ST3687. *K. michiganensis* and *K. oxytoca* strains could not be assigned an ST type. Based on Kleborate analysis, the 31 ESBL‐producing *Klebsiella* strains were classified into 13 different KL types: KL124, KL110, KL132, KL101, KL114, KL108, KL113, KL148, KL169, KL118, KL1, KL29, and KL55. Plasmid analysis revealed that the *bla*_CTX-M_ genes were mainly located on IncFIB(K) (58.1%, 18/31) and IncN3 (16.1%, 5/31), with additional associations with IncQ1, IncHI1, IncHI2, IncFII, and IncL plasmids. In addition to ESBL‐encoding genes, the isolates also carried sulfonamide resistance genes (*sul1*, *sul2*, and *sul3*), aminoglycoside resistance genes (*aph*(*3"*)‐*Ib* and *aph*(*6*)‐*Id*), tetracycline resistance gene *tet*(A), and fosfomycin resistance gene *fosA6*. Notably, one *K. pneumoniae* strain carried *bla*_NDM−1_. Virulence gene analysis showed that, except for *K. michiganensis* and *K. oxytoca*, all 29 *K. pneumoniae* strains carried the virulence genes *mrkA* and *fimH* and the high virulence factor *iutA*. In addition, the colibactin gene *clpk1* was also detected in 7 *K. pneumoniae* strains (22.6%). According to the Sankey diagram (Figure 2), *K. pneumoniae* mainly carried *bla*_CTX-M−3_ and *bla*_CTX-M−15_, whereas one *K. oxytoca* strain carried *bla*_CTX-M−27_. IncFIB(K) and IncN3 were commonly associated with *K. pneumoniae* carrying *bla*_CTX-M−3_ and *bla*_CTX-M−15_, while IncHI2 is associated with *bla*_CTX-M−27_. Additionally, ST107 and ST716 were mainly associated with strains carrying *bla*_CTX-M−3_ and *bla*_CTX-M−15_.

Among the 31 ESBL‐producing *Klebsiella* isolates, resistance to multiple antimicrobial agents was observed, accompanied by a diverse array of resistance genes. To investigate the potential relationship between ARGs and ISs, we constructed a network diagram (Figure S8). The analysis revealed distinct correlations between certain ARGs and specific ISs. Notably, *bla*_TEM−1B_, *tet*(A), and *floR* were strongly associated with IS*6100*, while *sul1* and *qacE* showed a close relationship with the same element, IS*6100*. Additionally, *bla*_CTX-M−3_ and *bla*_CTX-M−15_ were predominantly correlated with IS*Ecp1*. These findings are consistent with the frequent detection of these resistance genes in the 31 ESBL‐producing *Klebsiella* isolates, suggesting that these ISs may play a role in the distribution and mobilization of the associated resistance genes.

### 3.5. Phylogenetic Analysis of ESBL‐Producing Strains

**Figure 3:**
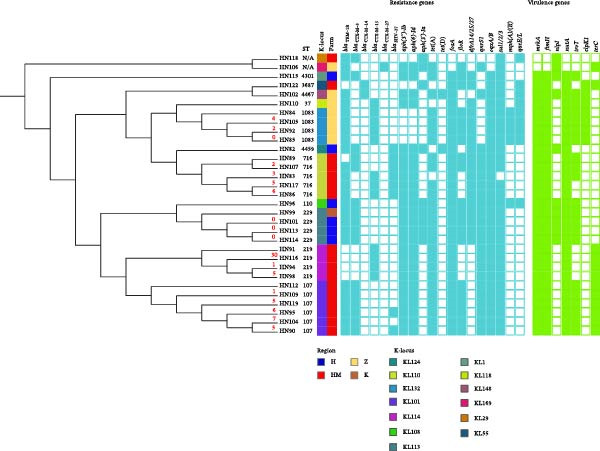
Phylogenetic analysis and basic characteristics of ESBL‐positive strains. The phylogenetic tree was constructed using Roary and FastTree based on SNPs in the core genome.

To investigate the phylogenetic relationships among the 31 ESBL‐producing *Klebsiella* strains isolated from dairy cows in different regions of Henan Province, a phylogenetic tree was constructed based on core‐genome SNPs. Previous studies have suggested that, for *Enterobacteriales*, isolates differing by ≤23 SNPs can be considered clonally related [23, 24]. The phylogenetic tree revealed that all *Klebsiella* isolates clustered within a single main clade, which further divided into several distinct sub‐branches (Figure 3). SNP analysis revealed that the ST1083 isolates (HN84, HN105, HN92, and HN85) clustered closely together, exhibiting minimal genetic divergence (0–4 SNP differences). All four ST1083 isolates originated from the same farm (Farm Z) and were collected from Zhengzhou. Similarly, the ST716 isolates from farm HM in Zhengzhou (HN89, HN107, HN83, HN117, and HN86) also formed a tight cluster, exhibiting 2–6 SNP differences. Another closely related cluster comprised ST107 isolates (HN112, HN109, HN119, HN95, HN104, and HN90) from farm HM in Zhengzhou, demonstrating SNP differences ranging from 1 to 7. In contrast, SNP analysis indicated that ST219 isolates from farm HM (HN116, HN94, and HN98) showed higher genetic diversity, with SNP differences ranging from 5 to 30. Notably, no SNP differences were observed among the ST229 isolates (HN99, HN101, HN113, and HN114), despite three of them (HN101, HN113, and HN114) being collected from farm H in Anyang and one (HN99) from farm K in Kaifeng, indicating high genetic homology across geographic regions.

### 3.6. Characteristics of A *bla*_NDM−1_‐Positive *K. pneumoniae* Strain

The *K. pneumoniae* strain HN148 possesses a 5,272,525 bp chromosome and two plasmids, one of which harbors the *bla*_NDM−1_ gene. ResFinder analysis revealed a wide array of resistance genes. On the chromosome, there is one β‐lactamase gene (*bla*_SHV−27_), two efflux pump genes (*oqxA* and *oqxB*), and the fosfomycin resistance gene, *fosA6*. Plasmid pHN148‐P1 carries no resistance genes, whereas the *bla*_NDM−1_‐positive plasmid pHN148‐NDM harbors six aminoglycoside resistance genes (*aac*(*3*)‐*IId*, *aph*(*6*)‐*Id*, *aadA16*, *aph*(*3′*)‐*Ia*, *aph*(*3″*)‐*Ib*, and *aph*(*6′*)‐*Ib-cr*), three folate‐pathway antagonist genes (*sul1*, *sul2*, and *dfrA27*), one rifampicin resistance gene (*arr-3*), and one chloramphenicol resistance gene (*catB3*).

**Figure 4:**
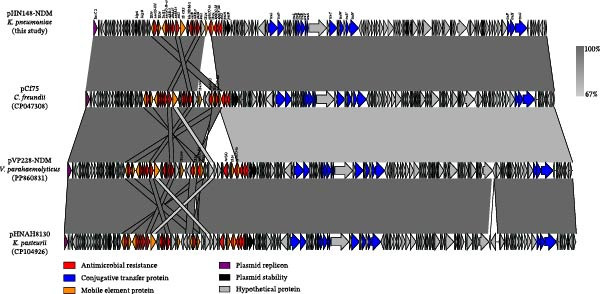
Structural comparison of *bla*_NDM−1_‐positive plasmid pHN148‐NDM with similar plasmids in GenBank. Black, blue, red, purple, orange, and gray arrows represent plasmid stability‐associated proteins, transfer‐related proteins, resistance genes, replicons, mobile elements, hypothetical elements, and unclassified elements, respectively. The arrows indicate the direction of transcription.

Plasmid pHN148‐P1 is 132,553 bp in length, has a GC content of 50.6%, contains 389 ORFs, and belongs to the IncFIB(K) group. The *bla*_NDM−1_‐positive plasmid pHN148‐NDM is 141,407 bp, with a GC content of 51.6%, and contains 409 ORFs. It belongs to the IncC 2‐type plasmid. Comparative genomic analysis showed that pHN148‐NDM shares complete sequence identity (100%) with plasmid pCf75 (CP047308.1, 146,252 bp) from *Enterobacter cloacae* isolated from human urine, with a 99% coverage. It also exhibits high similarity with plasmid pVP228‐NDM (PP860831.1, 157,661 bp) from *V. parahaemolyticus* isolated from shrimp. Conjugation experiments showed that pHN148‐NDM could be transferred to *E. coli* C600 at a frequency of 4.7 × 10^−5^. Sequence analysis of these plasmids revealed that they share a common backbone, including 14 *tra* genes (Figure 4). The pHN148‐NDM plasmid appears to be a descendant of pCf75, having replaced the IS*26*‐*aph*(*3′*)‐*Ia* region with IS*6100*‐*mph*(*A*)‐IS*26*‐*aac*(*3′*)‐*IId*. Similarly, the pVP228‐NDM plasmid likely evolved from pCf75 through an inversion rearrangement of the IS*26*‐*aac*(*3′*)‐*IId*‐IS*26* region, which also acquired a 10,522 bp fragment containing *tet*(A), IS*26*, and *aph*(*3′*)‐*Ia*. These findings suggest that plasmids carrying *bla*_NDM−1_ have the potential to disseminate across different bacterial species, genera, and even ecological niches, raising significant public health concerns.

## 4. Discussion

In this study, 174 bacterial strains were isolated from milk samples of dairy cows with mastitis in Henan Province, of which 65 (37.4%) were identified as *Klebsiella* species. In China, *Klebsiella* has been recognized as a major opportunistic pathogen in dairy herds, accounting for 20%–30% of cases [25, 26]. Among the 65 *Klebsiella* isolates, 61 belonged to the *K. pneumoniae* species complex (KpSC), with the KpI phylogroup predominating (93.8%, 61/65), consistent with its status as the principal lineage in both human and animal infections [27]. These 61 *K. pneumoniae* strains belonged to 24 different sequence types (STs), without any single ST dominating. This high ST diversity suggests a polyclonal infection pattern, likely driven by environmental reservoirs such as bedding, water, or feed. Such reservoirs may facilitate sporadic introductions into dairy herds rather than clonal outbreaks [28]. Notably, ST37 was detected in five strains (7.7%). This international high‐risk clone is frequently linked to extended‐spectrum β‐lactamase (ESBL) production and carbapenem resistance in human infections [29, 30], and its global distribution across human, animal, and environmental sources suggests the potential for cross‐regional and cross‐species transmission [31]. Capsule (KL) types among bovine *K. pneumoniae* were highly diverse, with no dominant subtype. This broad K‐locus variability contrasts sharply with human hypervirulent strains, where KL1 and KL2 predominate, conferring enhanced virulence via evasion of phagocytosis and complement‐mediated killing [32]. The observed KL diversity in bovine strains may reflect selective pressures within the mammary gland environment, favoring capsule switching as an adaptive mechanism for persistent infection. In contrast, the conserved KL types in human hvKp likely facilitate systemic dissemination [33]. O‐antigen diversity was comparatively lower, with six types being detected. O1 was the most common (47.5%, 29/61), followed by O3/O3a (19.7%, 12/61). O1 is prevalent in both human and bovine *K. pneumoniae* and is associated with serum resistance and biofilm formation. Its higher frequency in bovine strains may indicate host‐specific adaptation for udder colonization [34, 35].

Phylogenetic analysis divided the bovine *K. pneumoniae* strains into two major subclades, each showing distinct levels of genetic homology, with minimal SNP variation observed only among a few closely related isolates. This pattern suggests that the strains originated from multiple ancestral sources and are undergoing ongoing diversification, likely driven by introductions from different environmental or animal reservoirs. Plasmid replicon typing revealed multiple variants, predominantly IncFIB(K), IncFII(K), and IncHI1B—replicons frequently linked to MDR and the horizontal transfer of virulence genes in *K. pneumoniae*—potentially enhancing survival under antibiotic pressure in dairy farming environments [36–38]. Comparison with human‐origin hypervirulent *K. pneumoniae* (hvKp) strains revealed marked genetic and functional divergence. Large SNP distances indicate a distant evolutionary relationship at the core‐genome level, likely shaped by long‐term host‐specific adaptation and ecological separation. In bovine strains, SNP accumulation may reflect selective pressures in the mastitis niche—such as immune defense and nutrient limitation—leading to genetic trajectories distinct from those of human hvKp, which are adapted for systemic infections like liver abscess or sepsis [27, 39]. These evolutionary differences may also involve point mutations or insertion/deletion events in regulatory genes that down‐regulate hypervirulence‐associated traits [40, 41]. A notable difference is the complete absence of the *rmpA* gene in all bovine strains. In human hvKp, *rmpA*—typically carried on virulence plasmids—regulates the hypermucoviscous phenotype (positive string test) and drives invasive diseases such as pyogenic liver abscess [33, 42]. Its absence in bovine strains suggests a reduced hypervirulence potential, predisposing these bacteria to localized mastitis rather than systemic dissemination. This reflects an ecological partition: human hvKp strains prioritize virulence plasmid acquisition for metastatic spread, whereas bovine strains appear to favor MDR traits, likely selected via horizontal gene transfer under sustained antimicrobial use in farm settings [43–45]. Consistent with this partitioning, our bovine ST37 isolates were also highly divergent from human‐derived ST37 genomes, providing no evidence for recent sharing of this lineage between bovine and currently sampled human populations. Collectively, these findings define the epidemiological profile of bovine *K. pneumoniae* in China as characterized by high genetic and antigenic diversity, a dominance of MDR, and virulence features distinct from those of human hvKp.

Given the genetic diversity and MDR profiles observed, we further investigated the prevalence and molecular characteristics of extended‐spectrum β‐lactamase (ESBL) genes among these bovine *Klebsiella* isolates to better understand their role in antibiotic resistance dissemination. Among the 65 *Klebsiella* strains, ESBL genes were detected in 31 strains (47.7%). Compared with previous studies of milk samples from both healthy and mastitic cows [46–48], mastitic cow samples generally show higher rates of ESBL‐producing bacteria. This further underscores the direct association between antimicrobial usage and the prevalence of ESBL in dairy herds. Notably, mastitis is typically associated with pathogens such as *S. aureus*, *S. agalactiae*, and *E coli*, whereas *K. pneumoniae*‐associated mastitis is reported less frequently. In our study, *K. pneumoniae* had the highest detection rate, while the detection rates of other *Klebsiella* types were much lower. *K. michiganensis*, which is commonly isolated from plants, was also detected, suggesting possible introduction via contaminated feed [49]. These findings suggest that mastitic cows may act as reservoirs for multidrug‐resistant *Klebsiella* strains, particularly those harboring ESBL genes.

Among the ESBL‐producing *Klebsiella* strains, 80.6% carried multiple β‐lactamase genes, with *bla*_CTX-M_ being the most prevalent, followed by *bla*_TEM_. The dominance of the *bla*_CTX-M_ gene, particularly *bla*_CTX-M−3_ and *bla*_CTX-M−15_, mirrors global trends [50] and supports findings that *bla*_CTX-M_ variants are becoming the predominant ESBL genes in *Enterobacteriaceae* [51, 52]. Several strains also carried other resistance genes, such as *tet*(A) and *floR*, which may further enhance their MDR and environmental fitness [53, 54]. Importantly, all ESBL‐producing *Klebsiella* isolates remained sensitive to meropenem, which is considered a last‐resort option for treating infections caused by multidrug‐resistant Gram‐negative bacteria, including ESBL producers [55]. This result was expected as carbapenem antibiotics are not approved for use in dairy cows or other food animals in China [56]. Furthermore, drug usage records indicate that the widespread use of antibiotics, particularly third‐generation cephalosporins, may be a selective pressure driving the emergence of ESBLs and multidrug‐resistant bacteria on dairy farms. In recent years, ESBL‐producing *Enterobacteriaceae* isolates have spread from hospitals to the community and environment, being isolated from various sources, including cows, chickens, pigs, raw milk, and freshwater environments [57–60]. Therefore, continuous monitoring of ESBL‐producing strains across animals, humans, and the environment is necessary, alongside investigations into the factors influencing their selection and transmission.

In *Enterobacteriaceae*, ESBL genes are primarily associated with mobile genetic elements (MGEs), such as plasmids, ISs, transposons, integron cassettes, and phages that mediate intra‐ and intercellular mobility [61–63]. MGEs play a crucial role in the dissemination of ESBL genes. Minimal SNP variation within identical sequence types (STs) suggests that clonal transmission of ESBL‐producing *Klebsiella* occurs within the same ST. In contrast, substantial SNP differences among isolates of distinct STs indicate horizontal dissemination of ESBL genes mediated predominantly by plasmids. Indeed, genetic localization revealed that the majority of ESBL genes, particularly *bla*_CTX-M−3_ and *bla*_CTX-M−15_, are located on plasmids. This finding suggests that plasmids, especially IncFIB(K) and IncN3 types, possibly contribute significantly to the spread of CTX‐M genes across different *Klebsiella* STs through conjugation. Previous studies have similarly highlighted the global distribution of ESBL variants associated with IncF and IncN plasmids, emphasizing their roles in horizontal gene transfer [64–66]. Notably, IncF plasmids often carry additional genes related to bacterial fitness, virulence, and other ARGs, further promoting bacterial survival and spread in various hosts [67, 68]. Collectively, these results highlight the critical role of MGEs and specific plasmid types in facilitating the dissemination of ESBL genes in dairy farm environments.

In addition to ESBL genes, the *bla*_NDM−1_ gene was identified in one *K. pneumoniae* isolate, raising concern due to its broad‐spectrum carbapenem resistance. The *bla*_NDM−1_ gene is commonly located on various plasmid types, including mobilizable plasmids such as IncX3, IncFII, and IncC 2‐type plasmids, as well as plasmids of unknown type [69–72]. These plasmids conjugate across bacterial hosts, driving horizontal transfer of *bla*_NDM−1_ and boosting carbapenem resistance [73]. In this study, the *bla*_NDM−1_‐positive plasmid pHN148‐NDM belongs to the IncC 2‐type plasmid, a major global vector for NDM dissemination [74]. This plasmid harbors multiple ARGs arranged in resistance islands (AMR islands), facilitating horizontal gene transfer among bacteria. These AMR islands are classified into two subgroups, ARI‐A, which typically carries more resistance genes and has a broad host range facilitating transfer across diverse bacterial species, and ARI‐B, which is more restricted, generally spreading within specific species or strains [75]. Comparative genomic analysis revealed that pHN148‐NDM shares a conserved backbone with plasmids from various sources, including shrimp‐derived *V. parahaemolyticus* pVP228‐NDM and human‐derived *Enterobacter cloacae* pCf75. Evolutionary analyses suggest that recombination events, insertion sequence rearrangements (e.g., IS*26* and IS*6100*), and acquisition of additional resistance genes such as *mph*(A) and *tet*(A) contribute to the adaptability, mobility, and persistence of these plasmids [76]. Structural variations, including inversion rearrangements and gene cluster substitutions, further demonstrate their genetic plasticity and potential for rapid dissemination. Collectively, these findings emphasize the significant threat posed by *bla*_NDM−1_‐bearing plasmids in facilitating horizontal gene transfer across bacterial species and ecological niches, challenging global antimicrobial resistance management.

## 5. Conclusions

This study reveals high genetic and antigenic diversity alongside MDR among bovine‐origin *Klebsiella* strains from mastitic dairy cows in Henan Province. The population was dominated by the KpI phylogroup with diverse sequence types, indicating multiple sources of introduction and a distant evolutionary relationship with human hypervirulent *K. pneumoniae*. Although key virulence genes like *rmpA* were absent, there was a high prevalence of ESBL‐producing strains, with *bla*_CTX-M_ genes primarily spread via IncFIB(K) and IncN3 plasmids. The detection of a *bla*_NDM−1_‐carrying IncC 2‐type plasmid highlights the emergence of carbapenem resistance in livestock environments. These findings underscore the urgent need for improved antimicrobial stewardship and ongoing surveillance across the animal–human–environment interface to mitigate public and animal health risks.

## Funding

This work was supported by the National Natural Science Foundation of China (Grant 32273065), the Henan Province Outstanding Youth Science Fund Project (Grant 242300421109), and the Henan Province Key Research and Development Project‐Key Project of International Science and Technology cooperation of Henan Province (Grant 2411115211000).

## Ethics Statement

This study did not involve animal experimentation. Milk samples were collected from cows with mastitis on dairy farms with permission from the farm owners; therefore, formal animal ethics approval was not required.

## Conflicts of Interest

The authors declare no conflicts of interest.

## Supporting Information

Additional supporting information can be found online in the Supporting Information section.

## Supporting information


**Supporting Information** Table S1: Primers used in this study. Table S2: Drug resistance spectrum of 31 ESBL‐producing *Klebsiella* strains. Figure S1: Detection rates of antibiotic resistance genes in 65 *Klebsiella* strains. Figure S2: Capsular types of 61 *K. pneumonias* strains. Figure S3: Types of lipopolysaccharides in 61 *K. pneumonias* strains. Figure S4: 61 KpI‐type *K. pneumoniae* strains clonal subpopulations. Figure S5: Phylogenetic tree and distribution of sequence types, capsule types, antigens, plasmids, and virulence genes among 61 bovine‐origin *K. pneumoniae* strains. Figure S6: Antibiotic resistance rates of 31 ESBL‐producing *Klebsiella* strains to 14 antibiotics. Figure S7: Distribution of the number of antibiotic resistances among ESBL‐producing *Klebsiella* strains. Figure S8: Network diagram illustrating the co‐occurrence patterns between ARGs and ISs. Nodes represent ARGs (in red) or ISs (in black). Lines connecting the nodes indicate relationships between them, with numbers on the lines representing correlation coefficients between paired nodes.

## Data Availability

The data will be made available upon request.
