# Supplementary material for: Epidemiological and Genomic Characteristics of bla CTX-M and bla NDM‐Producing K. pneumoniae From Dairy Cows in Henan Province, China
Source: Transbound Emerg Dis. 2026 May 30;2026:5331014. doi: 10.1155/tbed/5331014 (PMC13239034; doi:10.1155/tbed/5331014)
Supplement: Supplementary file 1 — Supporting Information Table S1: Primers used in this study. Table S2: Drug resistance spectrum of 31 ESBL‐producing Klebsiella strains. Figure S1: Detection rates of antibiotic resistance genes in 65 Klebsiella strains. Figure S2: Capsular types of 61 K. pneumonias strains. Figure S3: Types of lipopolysaccharides in 61 K. pneumonias strains. Figure S4: 61 KpI‐type K. pneumoniae strains clonal subpopulations. Figure S5: Phylogenetic tree and distribution of sequence types, capsule types, antigens, plasmids, and virulence genes among 61 bovine‐origin K. pneumoniae strains. Figure S6: Antibiotic resistance rates of 31 ESBL‐producing Klebsiella strains to 14 antibiotics. Figure S7: Distribution of the number of antibiotic resistances among ESBL‐producing Klebsiella strains. Figure S8: Network diagram illustrating the co‐occurrence patterns between ARGs and ISs. Nodes represent ARGs (in red) or ISs (in black). Lines connecting the nodes indicate relationships between them, with numbers on the lines representing correlation coefficients between paired nodes. [file TBED-2026-5331014-s001.docx]

| Gene name | Primer name | Primer Sequences | Fragment length |
| --- | --- | --- | --- |
| *16S rRNA* | 16S-F 16S-R | AGAGTTTGATCCTGGCTCAG ACGGCTACCTTGTTACGACTT | 1464bp |
| *floR* | *floR*-F *floR*-R | GTATGGGCACCTTCTTCGTCT CAGCCCCAACGAAACCAGT | 494bp |
| *tet*(A) | *tet*(A)-F *tet*(A)-R | CCTCCTGCGCGATCTGGTTC TCCTCGCCGAAAATGACCCAA | 627bp |
| *bla*_NDM_ | *bla*NDM-F *bla*NDM-R | CCCGGTCGCGAAGCTGAGCA CAGGCAGCCACCAAAAGCGAT | 603bp |
| *bla*_CTX-M_ | *bla*CTX-F *bla*CTX-R | ATGTGCAGTACCAGTAAAGT  TGGGTRAAGTARGTCACCAGAA | 593bp |
| *bla*_OXA_ | *bla*_OXA_-F *bla*OXA-R | GAGTATCGCAATTTTCGCC  GTGTTCAGCGTTGTTTGC | 450bp |

**Table S1：**Primers used in this study.

**Table S2:** Drug resistance spectrum of 65 *Klebsiella* strains.

| Number of drug resistance | Number of strains | Drug resistance spectrum |
| --- | --- | --- |
| 0 | 4 | / |
| 1 | 22 | AMP |
| 1 | 4 | DOX |
| 2 | 1 | AMP, FOX |
| 2 | 1 | AMP, COL |
| 2 | 1 | AMP, FFC |
| 2 | 1 | AMP, DOX |
| 4 | 2 | FUR, CTX, AMP, CAZ |
| 4 | 1 | ENR, FUR, CTX, AMP |
| 5 | 1 | FFC, FUR, CTX, AMP, DOX |
| 5 | 1 | FUR, CTX, AMP, DOX, CAZ |
| 5 | 1 | ENR, FUR, CTX, AMP, DOX |
| 5 | 1 | FFC, FUR, CTX, AMP, GEN |
| 6 | 4 | ENR, FUR, CTX, AMP, DOX, CAZ |
| 6 | 1 | ENR, FUR, CTX, AMP, DOX, GEN |
| 6 | 4 | FFC, ENR, FUR, CTX, AMP, DOX |
| 6 | 1 | FFC, FUR, CTX, AMP, DOX, CAZ |
| 6 | 1 | FFC, FUR, CTX, AMP, DOX, FOX |
| 7 | 3 | FFC, ENR, FUR, CTX, AMP, DOX, CAZ |
| 7 | 1 | FFC, ENR, FUR, CTX, AMP, DOX, GEN |
| 7 | 1 | ENR, COL, FUR, CTX, AMP, DOX, CAZ |
| 7 | 1 | FFC, FUR, CTX, AMP, DOX, FOX, CAZ |
| 7 | 1 | FUR, CTX, AMP, GEN, FOX, CAZ, MEM |
| 8 | 1 | CIP, FFC, ENR, FUR, CTX, AMP, DOX, CAZ |
| 8 | 1 | FFC, ENR, FUR, CTX, AMP, DOX, CAZ, TGC |
| 8 | 1 | FFC, ENR, FUR, CTX, AMP, DOX, GEN, TGC |
| 9 | 1 | CIP, FFC, ENR, FUR, CTX, AMP, DOX, CAZ, TGC |
| 9 | 1 | CIP, FFC, ENR, FUR, CTX, AMP, DOX, GEN, CAZ |
| 10 | 1 | CIP, FFC, ENR, COL, FUR, CTX, AMP, DOX, CAZ, TGC |

Note: AMP, ampicillin; CTX, cefotaxime; CAZ, Ceftazidime; FFC, Florfenicol; COL, Colistin; FOX, cefoxitin; TGC, Tigecycline; DOX, doxycycline; GEN, gentamicin; ENR, enrofloxacin; CIP, ciprofloxacin; MEM, meropenem; FUR, cefuroxime. /, Non-resistant phenotype.


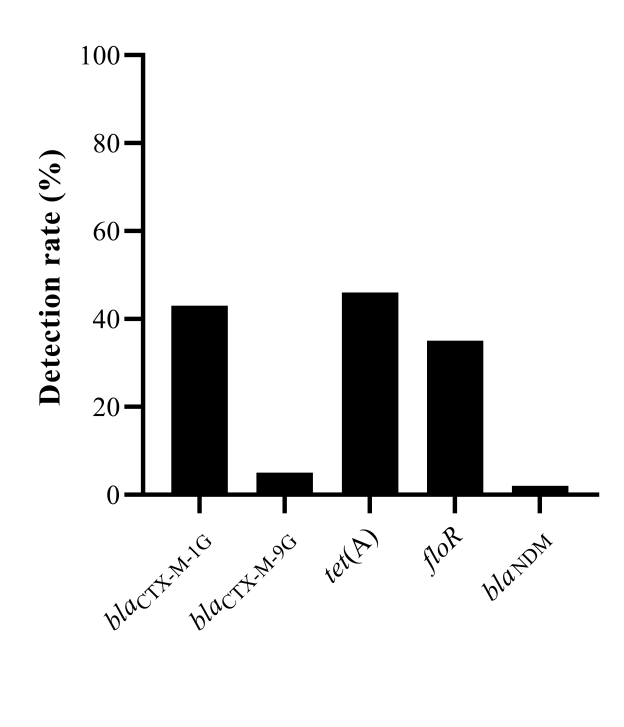


**Figure S1:** Detection rates of antibiotic resistance genes in 65 *Klebsiella* strains.


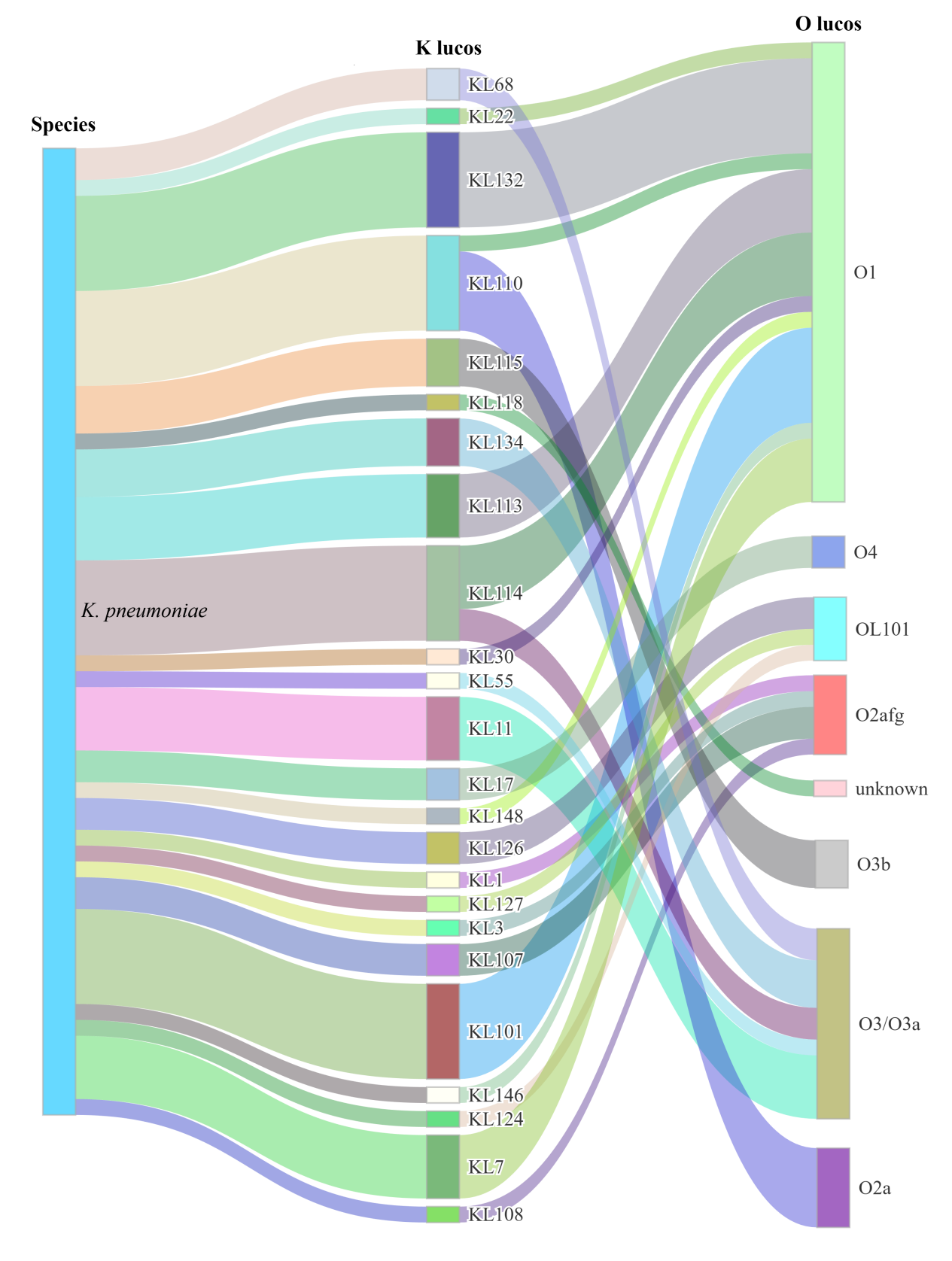


**Figure S2:** Distribution of capsular K-locus (KL) and O-antigen types among 61 *K. pneumoniae* isolates. “unknown” indicates isolates not confidently assigned.


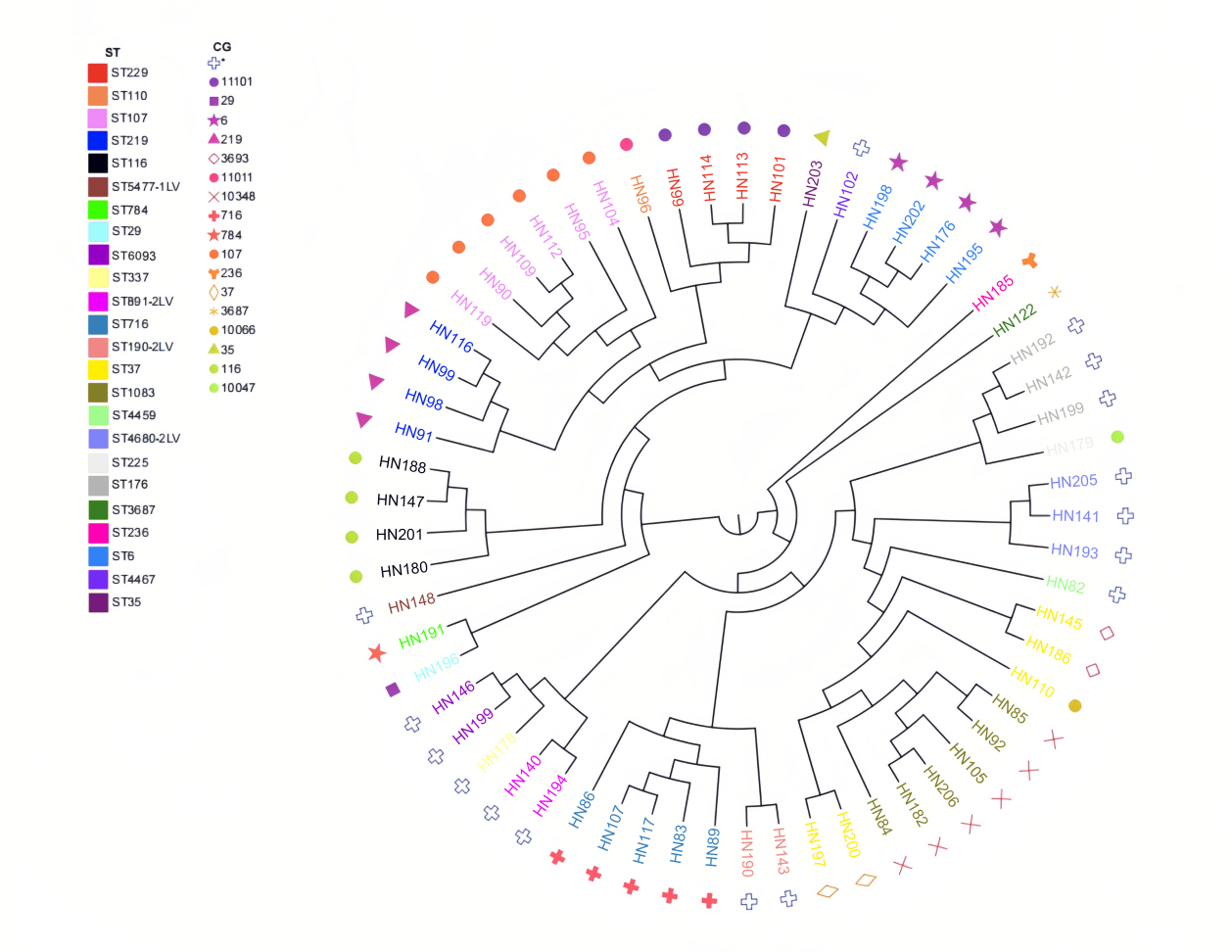


**Figure S3:** Phylogenetic relationships and basic characteristics of 61 bovine-origin *K. pneumoniae* isolates.


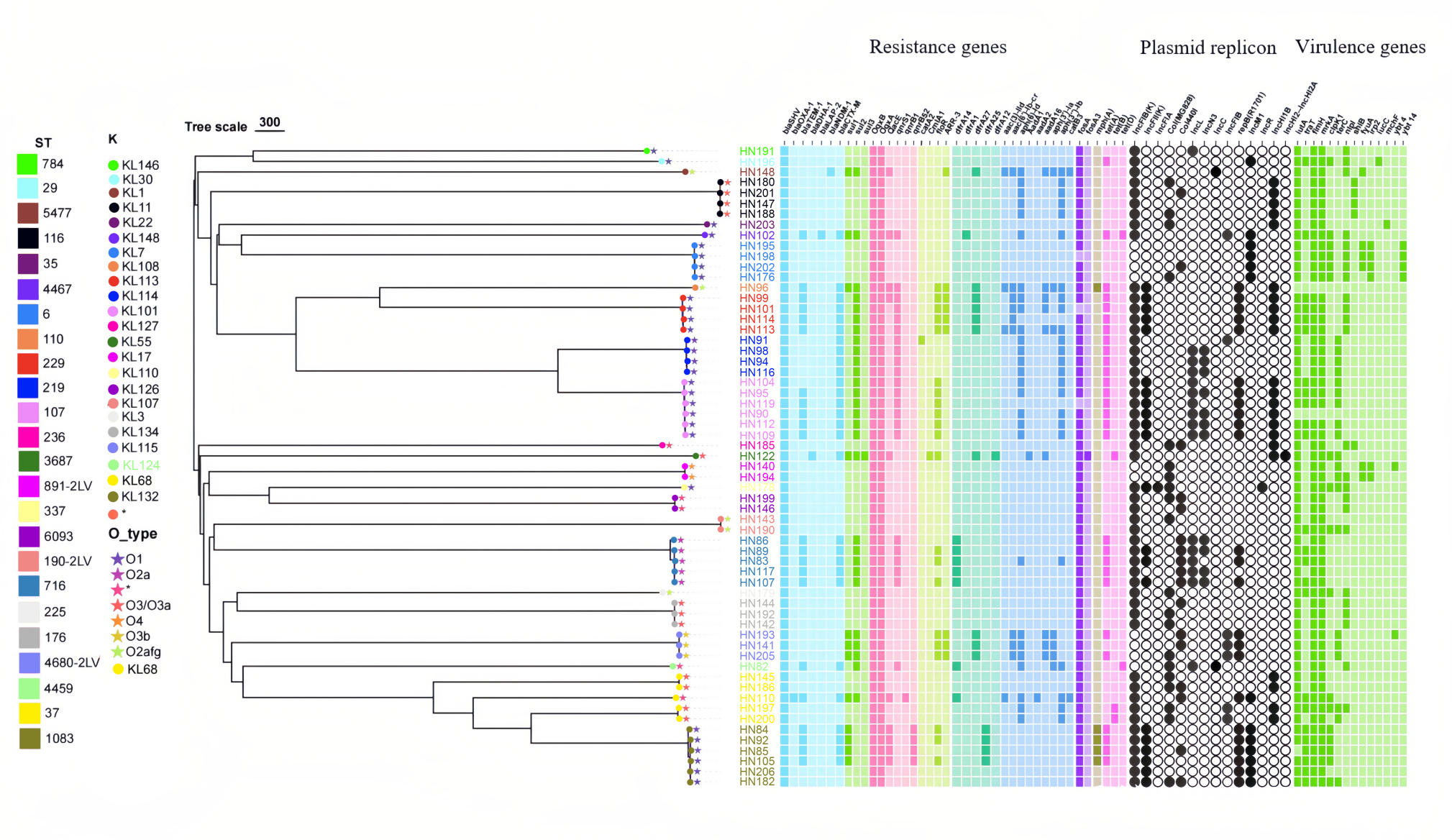


**Figure S4:** Phylogenetic tree and distribution of sequence types, capsule types, antigens, plasmids, and virulence genes among 61 bovine-origin *K. pneumoniae* strains.


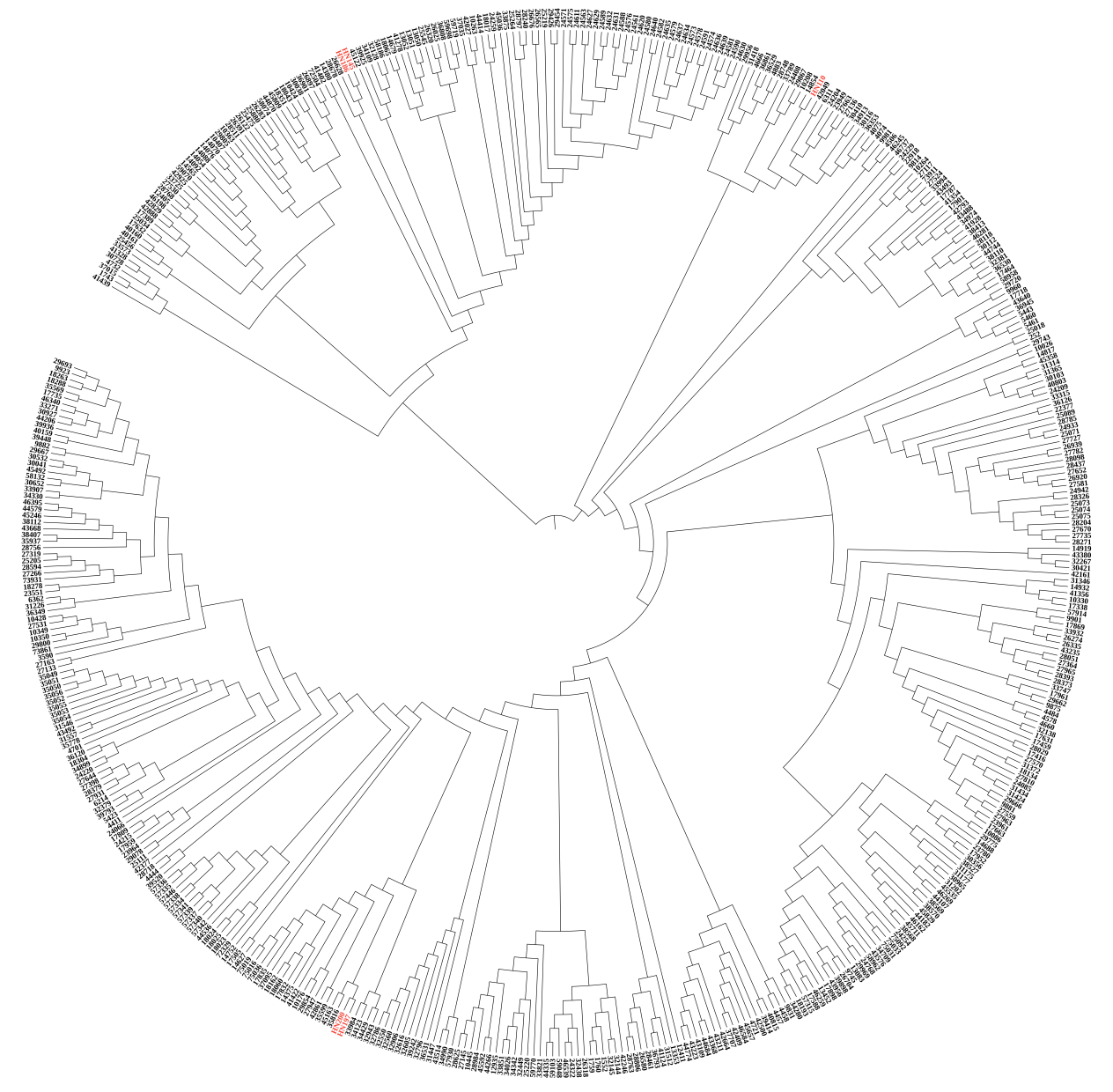


**Figure S5:** Core-genome SNP phylogeny of ST37 *K. pneumoniae* from bovine mastitis and human-derived genomes.


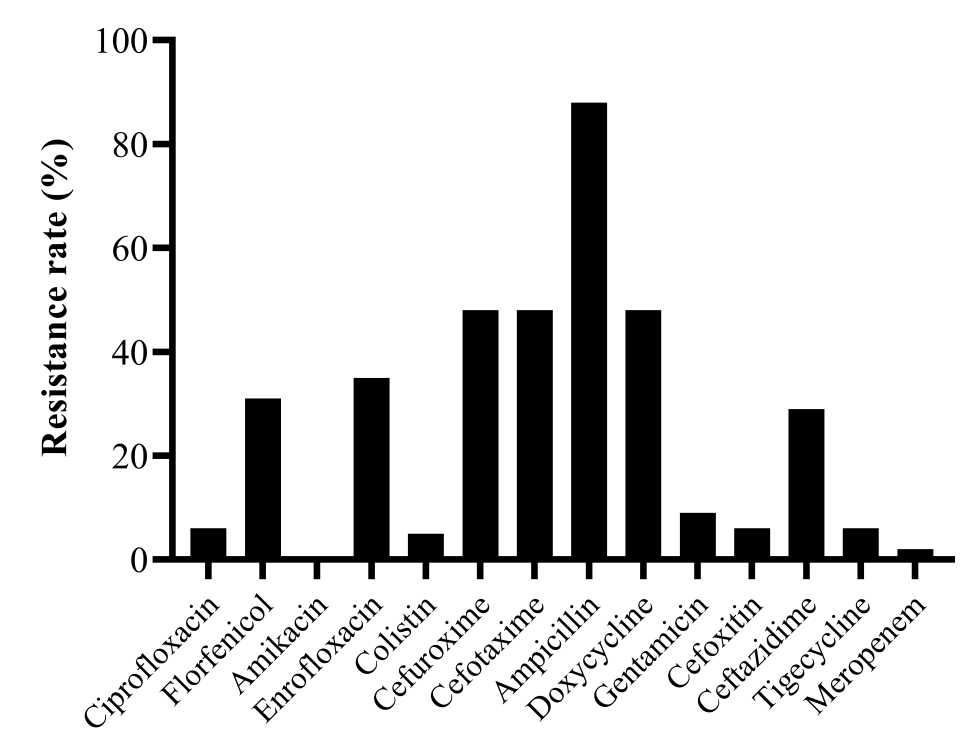


**Figure S6:** Antibiotic resistance rates of 65 *Klebsiella* strains to 14 antibiotics.


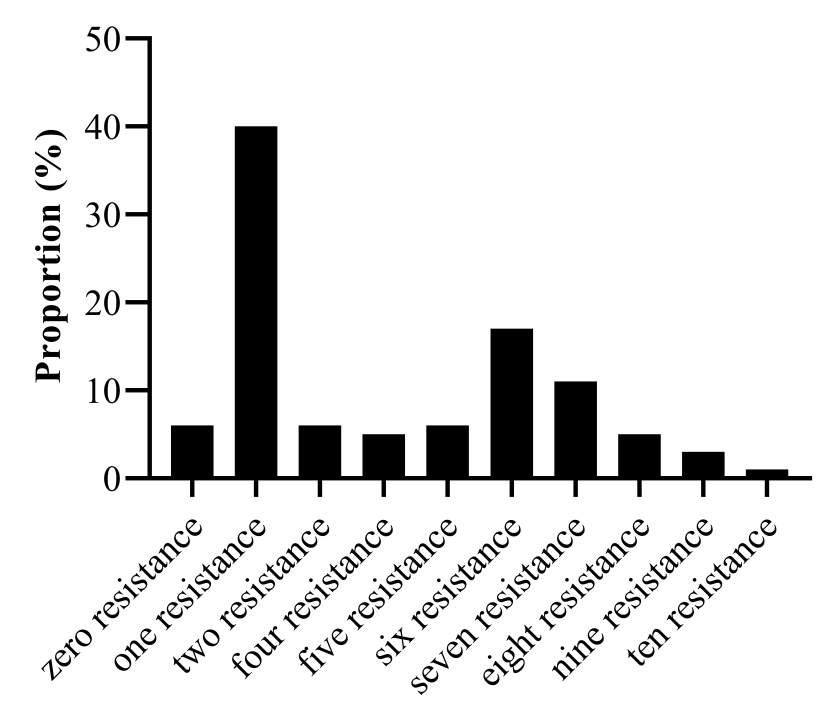


**Figure S7:** Distribution of the number of antibiotic resistances among 65 *Klebsiella* strains.


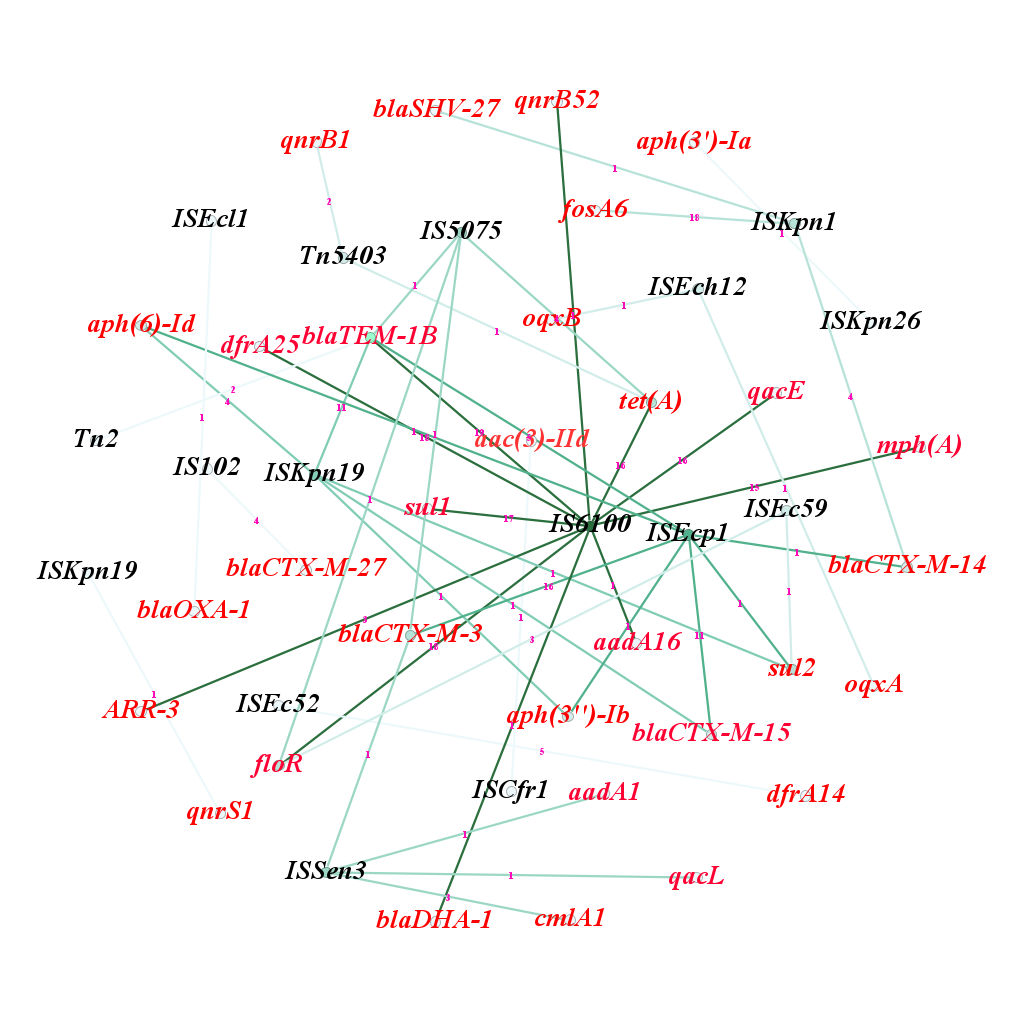


**Figure S8: Network diagram illustrating the co-occurrence patterns between ARGs and ISs.** Nodes represent ARGs (in red) or ISs (in black). Lines connecting the nodes indicate relationships between them, with numbers on the lines representing correlation coefficients between paired nodes.
